# Primary Hyperparathyroidism: 18F-Fluorocholine PET/CT vs. 4D-CT for Parathyroid Identification: Toward a Comprehensive Diagnostic Framework—An Updated Review and Recommendations

**DOI:** 10.3390/jcm14155468

**Published:** 2025-08-04

**Authors:** Gregorio Scerrino, Nunzia Cinzia Paladino, Giuseppa Graceffa, Giuseppina Melfa, Giuseppina Orlando, Renato Di Vuolo, Chiara Lo Cicero, Alessandra Murabito, Stefano Radellini, Pierina Richiusa, Antonio Lo Casto

**Affiliations:** 1 Unit of Endocrine Surgery, Department of Surgical Oncological and Oral Sciences, Policlinico “P. Giaccone”, University of Palermo, Via Liborio Giuffré 5, 90127 Palermo, Italy; 2 Department of General and Endocrine Surgery, Conception Hospital, Aix-Marseille University, 147 Boulevard Baille, 13005 Marseille, France; nunzia.paladino@ap-hm.fr; 3 Unit of General and Oncology Surgery, Department of Surgical Oncological and Oral Sciences, Policlinico “P. Giaccone”, University of Palermo, Via Liborio Giuffré 5, 90127 Palermo, Italy; giuseppa.graceffa@unipa.it; 4 Unit of General and Emergency Surgery, Department of Surgical Oncological and Oral Sciences, Policlinico “P. Giaccone”, University of Palermo, Via Liborio Giuffré 5, 90127 Palermo, Italy; giuseppina.melfa@policlinico.pa.it (G.M.); giusi_orlando@hotmail.it (G.O.); divuolorenato@gmail.com (R.D.V.); chiaralocicero03@gmail.com (C.L.C.); 5 Unit of Nuclear Medicine, Biomedical Department of Internal and Specialist Medicine, Policlinico “P. Giaccone”, University of Palermo, 90127 Palermo, Italy; alessandra.murabito@policlinico.pa.it; 6 Section of Endocrinology, Department of Health Promotion Sciences Maternal and Infantile Care, Internal Medicine and Medical Specialties (PROMISE), University of Palermo, 90127 Palermo, Italy; radellinistefano@gmail.com (S.R.); pierina.richiusa@policlinico.pa.it (P.R.); 7 Section of Radiology, Department of Biomedicine, Neuroscience and Advanced Diagnostics (BIND), Policlinico “P. Giaccone”, University of Palermo, 90127 Palermo, Italy; antonio.locasto@unipa.it

**Keywords:** primary hyperparathyroidism (pHPT), 18F-fluorocholine PET/CT (FCH-PET), 4D-CT, preoperative imaging, minimally invasive surgery

## Abstract

**Introduction**: Primary hyperparathyroidism (pHPT) is an endocrine disorder characterized by excessive parathyroid hormone production, typically due to adenomas, hyperplasia, or carcinoma. Preoperative imaging plays a critical role in guiding surgical planning, particularly in selecting patients for minimally invasive procedures. While first-line imaging techniques, such as ultrasound and 99mTc-sestamibi scintigraphy, are standard, advanced second-line imaging modalities like 18F-fluorocholine PET/CT (FCH-PET) and four-dimensional computed tomography (4D-CT) have emerged as valuable tools when initial diagnostics are inconclusive. **Methods**: This article provides an updated review and recommendations of the role of these advanced imaging techniques in localizing parathyroid adenomas. **Results**: FCH-PET has shown exceptional sensitivity (94% per patient, 96% per lesion) and is particularly useful in detecting small or ectopic adenomas. Despite its higher sensitivity, it can yield false positives, particularly in the presence of thyroid disease. On the other hand, 4D-CT offers detailed anatomical imaging, aiding in the identification of parathyroids in challenging cases, including recurrent disease and ectopic glands. Studies suggest that FCH-PET and 4D-CT exhibit similar diagnostic performance and could be complementary in preoperative planning of most difficult situations. **Conclusions**: This article also emphasizes a multimodal approach, where initial imaging is followed by advanced techniques only in cases of uncertainty. Although 18F-fluorocholine PET/CT is favored as a second-line option, 4D-CT remains invaluable for its high spatial resolution and ability to guide surgery in complex cases. Despite limitations in evidence, these imaging modalities significantly enhance the accuracy of parathyroid localization, contributing to more targeted and minimally invasive surgery.

## 1. Introduction

Primary hyperparathyroidism (pHPT) is an endocrine disorder characterized by the overproduction of parathyroid hormone (PTH) leading to hypercalcemia [1]. The most common cause of pHPT is parathyroid adenomas, although carcinomas also contribute [2,3]. In contrast, parathyroid hyperplasia is mainly associated with secondary and tertiary hyperparathyroidism, and is not classified as a neoplastic lesion in the 2022 WHO classification, although it may occasionally be observed in hereditary forms of primary hyperparathyroidism and, more rarely, in sporadic cases [1,2,4]. The main treatment for pHPT is surgical removal of the affected parathyroid glands [2]. Preoperative imaging is crucial to guide the surgical approach, not so much for the indication itself but to transfer as many cases as possible to focused surgery in order to minimize its invasiveness and reduce complications [5]. Currently, ultrasounds and 99mTc-sestamibi scintigraphy are commonly regarded as first-line investigations, whereas 18F-fluorocholine PET/CT or four-dimensional CT(4D-CT) are considered second-line investigations. The cases where traditional, first-line imaging methods are inconclusive could become challenging, and the use of any of the second-line imaging tools is not yet well established because it depends on the experience of each center [6,7].

This article aims to evaluate the role of two advanced imaging modalities, PET with fluorocholine (18F-fluorocholine) (FCH-PET) and 4D-CT, in the preoperative localization of parathyroid adenomas.

By reviewing available literature, we will synthesize the most current evidence on their diagnostic accuracy and propose an updated algorithm for their use in clinical practice.

## 2. Methods

The literature review designed to fulfill the objectives of this study was conducted in two distinct phases. The MeSH terms ‘18F-fluorocholine PET-CT (FCH-PET)’ and ‘four-dimensional computed tomography (4D-CT)’ were individually combined with ‘hyperparathyroidism’ to retrieve relevant publications on these diagnostic techniques since their initial appearance in the scientific literature. Accordingly, studies on PET-choline were considered from 2014 onward, marking its first reported use in this context, while for 4D-CT, the search was extended back to 2006, the year of its earliest documented application.

Two independent researchers performed searches across PubMed, Web of Science, and Scopus, ensuring the removal of duplicate records. Only articles published in English were included. The extracted data covered publication year, study type, MeSH terms, and abstract content.

This selection criterion helped ensure a focus on the most up-to-date findings while excluding older studies that may no longer reflect current clinical perspectives.

Retrieved abstracts were systematically reviewed to assess relevance, followed by an in-depth evaluation of selected full-text articles. This critical analysis aimed to identify key arguments, emerging patterns, and novel insights.

By synthesizing the findings, the study sought to highlight areas of agreement, divergences, and significant contributions within the literature, ultimately providing a comprehensive understanding of how these imaging modalities assist in localizing hyperfunctioning parathyroid glands in primary hyperparathyroidism (HPT1).

## 3. Results

### 3.1. FCH-PET

The ability of FCH-PET to identify parathyroid hyperplasia was demonstrated completely by chance in 2014 [8]. Since then, a number of articles have appeared in the literature, aiming to further demonstrate this ability of a metabolite, originally developed for prostate neoplastic tissue, to identify its diagnostic accuracy and to specify its inclusion in a diagnostic algorithm. Thus, FCH-PET is a relatively recent functional imaging technique for the evaluation of hyperparathyroidism, with still limited yet expanding evidence in the literature. Parathyroid adenomas and hyperplastic glands exhibit increased cellular proliferation and metabolic activity, along with an upregulation of choline kinase, which enhances choline uptake. Leveraging this biological mechanism, 18F-fluorocholine, a radiolabeled choline analog, has been employed for the detection and characterization of parathyroid adenomas [9,10]. In recent years, a growing number of studies, many of which have been included in the present review, have confirmed the substantial interest this technique has generated in the localization of enlarged parathyroid glands.

In 2015, a pilot study involving 17 patients with various forms of hyperparathyroidism—primary, secondary, and lithium-induced—compared the diagnostic performance of 18F-fluorocholine PET/CT (FCH-PET), ultrasound (US), and dual-tracer 123I/99mTc-sestaMIBI dual-phase scintigraphy in the preoperative localization of pathological parathyroid glands. Ultrasound was performed using standard high-frequency linear probes by experienced operators, although the exact technical parameters were not specified. Scintigraphy involved both planar and SPECT/CT imaging, and the reported sensitivity of 58–83% reflects variability based on the acquisition protocol and the image interpretation method (planar vs. SPECT vs. dual-tracer subtraction). FCH-PET showed a sensitivity of 94% per patient and 96% per lesion, clearly outperforming both US (38%) and conventional nuclear imaging in this series [11].

Subsequent studies [12,13] have demonstrated the ability of FCH-PET to detect enlarged parathyroids even in cases of negative or equivocal conventional diagnostics, and even in the coexistence of multinodular goiter [14].

The excellent results shown so far subsequently led to FCH-PET being considered as a first-line imaging technique for localizing hyperfunctioning parathyroids in patients with [15]. However, this direction has not yet met with unanimous consensus.

However, this knowledge soon led to the method also being successfully employed in the hyperparathyroidism of uremic patients [16,17].

A systematic literature review by Evangelista et al. evaluated and compared the diagnostic accuracy of FCH-PET and PET/MRI in patients with primary hyperparathyroidism (pHPT), using conventional imaging modalities as a reference. The analysis included 23 studies and demonstrated that FCH-PET outperformed both ultrasound and MIBI/SPECT scintigraphy in terms of localization accuracy [18]. Additionally, PET/MRI proved to be more effective than MRI alone for detecting parathyroid lesions. Overall, the review confirmed that FCH-PET offers superior performance compared to conventional imaging techniques, with clinical utility in both first-time pHPT and recurrent pHPT [18].

More recently, numerous trials, which in several cases recruited large numbers of patients (>100), have shown that FCH-PET is a highly sensitive investigation, ranging from 76 [19] to 100% [20,21], with a lower error rate in differentiating superior from inferior parathyroids [22], much higher in detecting small adenomas than conventional diagnostics [23]. Other studies have shown that, although the diagnostic capacity is reduced in the presence of disease of the four glands, FCH-PET is highly sensitive when used as a second-level investigation, reducing the error in surgical indication by up to 2% [9]. Ultimately, FCH-PET has been shown, due to its high sensitivity, to greatly increase the percentage of patients who are candidates for minimally invasive techniques [24].

In recent years, several studies have confirmed a clear advantage of FCH-PET over [99mTc] sestamibi and ultrasonography [25,26,27,28]. For this reason, although some studies place this investigation at the second level [9], in others, FCH-PET has demonstrated excellent accuracy and could be considered the first-line examination for the localization of pathological parathyroids [29].

It has also been pointed out that, even in the presence of positive MIBI scintigraphy, this technique is still useful to improve localization for the purpose of minimally invasive surgery [30]. A recent study also aimed to compare the localization of hyperfunctioning parathyroids between experienced nuclear physicians and a deep learning model. However, this study concludes that, at present, FCH-PET is highly accurate for the localization of pathological parathyroids, whereas deep learning needs further improvement before it can be used in clinical practice [10].

### 3.2. 4D-CT

Four-dimensional computed tomography (4D-CT) relies on capturing images in three perpendicular planes—axial, coronal, and sagittal—across one non-contrast phase and three contrast-enhanced phases. The term ‘four-dimensional’ refers to the integration of contrast dynamics as an additional parameter. More recently, MRI has been explored as an alternative for 4D imaging of the parathyroid glands, though its effectiveness in this application remains under investigation [31].

Although experience with four-dimensional computed tomography (4D-CT) in the preparatory planning of parathyroidectomy was first published in 2006 [32], the studies on this subject were still limited 6 years later, so much so that, although a meta-analysis clearly showed the advantage of this investigation over ultrasound and Sestamibi-SPECT (with sensitivities of over 89% versus 76.1% and 78.9% respectively), this limitation was stated by the authors themselves [33]. At the same time, in an economic analysis of the use of 4D-CT in HPT1 surgery Abbott and coll (2012) pointed out that this diagnostic investigation allowed an increase in focused techniques from 80.5% to 90.3%, with a reduction of hospital days from 0.61 to 0.23. Despite the higher costs, 4D-CT therefore allowed a clear improvement in surgical strategies [34].

In a subsequent study carried out on a case history of non-localized hyperparathyroidism (negative ultrasound and Sestamibi), 4D-CT correctly identified the side of the neck: 76% and the correct quadrant: 49%. This allowed a unilateral approach to be performed in 72% of patients, reducing the surgical dissection [35].

In more recent studies, 4D-CT has also been shown to be effective in cases of primary hyperparathyroidism (pHPT) with low PTH levels. The sensitivity of the technique decreased from 94.7% for PTH > 100 pg/mL to 88% in patients with lower PTH levels, while still remaining within an acceptable range [36].

More recently, it has been reaffirmed that the high accuracy of 4D-CT in identifying the correct laterality facilitates a more frequent use of focused parathyroid surgery, with the potential benefits of reducing surgical invasiveness, shortening recovery times, and minimizing complications [37].

### 3.3. Comparative Studies of Diagnostic Performances

The first study designed to compare the diagnostic accuracy of FCH-PET and 4D-CT in the localization of both eutopic and ectopic parathyroid adenomas was published in 2017 [31]. This study included a small cohort of five patients diagnosed with primary hyperparathyroidism. All patients underwent FCH-PET, performed 60 min after the administration of 185 MBq of radiotracer. Within 2 weeks, a 4D-CT scan was conducted, incorporating three contrast phases (pre-contrast, arterial, and venous). In all cases, histopathological confirmation served as the diagnostic gold standard. It has been reported that there is a 100% concordance between the two imaging modalities, both of which identified seven lesions in five patients (four eutopic and three ectopic). Neither technique detected additional lesions beyond those visualized by the other, and all surgically excised lesions were confirmed as adenomas or hyperplasia. Although the limited sample size (only five patients) represents a significant weakness, the findings suggested that FCH-PET and 4D-CT demonstrated equivalent efficacy in the preoperative localization of pathological parathyroid glands. Notably, this study provided an early validation of 18F-fluorocholine PET/CT, which, at that time, had not yet been widely implemented in clinical practice.

A retrospective study involving 296 patients who underwent parathyroidectomy between 2010 and 2022 aimed to evaluate the diagnostic performance of various imaging modalities, including ultrasound (US), Sestamibi-SPECT, FCH-PET, and 4D-CT [38]. All patients initially underwent US and Sestamibi-SPECT as part of the standard diagnostic protocol; however, in 20 cases with inconclusive findings, FCH-PET was employed to improve localization accuracy. According to the results of this study, US correctly identified 77% of pathological parathyroid glands, while Sestamibi-SPECT demonstrated a detection rate of 68%. Among the 20 patients who underwent additional FCH-PET due to inconclusive first-line imaging, the technique yielded a reported sensitivity of 99%, confirming its superior diagnostic value in complex cases. Although 4D-CT was employed only in a limited number of challenging or recurrent cases, its diagnostic accuracy was estimated at 86%. These values are based on the metrics as reported in the original study, despite differences in terminology (i.e., identification rate, detection rate, sensitivity, and accuracy), which may reflect the specific methodology used for each modality in the referenced article. A limitation of this study is the relatively small sample size for patients undergoing 4D-CT, which restricts definitive conclusions on its general diagnostic performance.

A more recent study investigated the effectiveness of a combined diagnostic strategy in which FCH-PET was used as the primary imaging modality, followed by 4D-CT only in cases where PET/CT results were negative or inconclusive [39]. Conducted on a cohort of 166 patients who underwent surgery following imaging, the study assessed the diagnostic accuracy of each modality. PET/CT alone showed superior performance with a sensitivity of 83%, specificity of 97%, positive predictive value (PPV) of 90%, and negative predictive value (NPV) of 94%. In contrast, 4D-CT alone had lower diagnostic accuracy, with sensitivity at 53% and specificity at 84%. Nevertheless, when 4D-CT was added in cases with inconclusive PET/CT results, its sensitivity rose to 80%, specificity to 97% and overall diagnostic accuracy improved from 83% to 92%. This supports a tiered approach, with 4D-CT used selectively. Notably, this analysis referred specifically to FCH-PET/CT; FDG-PET/CT was not included, and its diagnostic value in pHPT is considered limited due to lower uptake in parathyroid tissue compared to 18F-fluorocholine. Similarly, 3D or non-contrast CT lacks the temporal perfusion data required for parathyroid characterization, and therefore cannot substitute for 4D-CT in this context.

A decision-model-based analysis was conducted to compare the clinical efficacy and cost-effectiveness of FCH-PET and 4D-CT in the preoperative localization of pathological parathyroid glands [40]. This model simulated clinical outcomes in a hypothetical cohort of 5000 patients with sporadic pHPT over a 21-year period, drawing from retrospective studies and systematic reviews. The analysis showed that 4D-CT was the most cost-effective imaging strategy, with a total cost of $10,276 per patient and 15.333 quality-adjusted life years (QALYs), offering a favorable balance between diagnostic accuracy and healthcare expenditure. In contrast, although FCH-PET exhibited greater diagnostic precision, it is 13% more expensive and its cost-effectiveness was lower, with a total cost of $11,619 per patient. For FCH-PET to be considered cost-effective within the U.S. healthcare framework, its cost per QALY gained would need to fall below $75,000. The study confirmed that 4D-CT remains a highly effective tool for identifying both single adenomas and multiglandular disease, especially when factoring in clinical and economic considerations. Nevertheless, when FCH-PET provides definitive localization, the incremental benefit of 4D-CT appears negligible. Overall, the findings support a personalized, stepwise diagnostic approach beginning with US and SPECT, followed by FCH-PET or 4D-CT as needed, reinforcing the importance of clinical guidelines that weigh accuracy and cost-effectiveness. Ultrasound and Sestamibi-SPECT, although less sensitive in difficult cases, are relatively inexpensive, widely available, and non-invasive, which justifies their continued role as first-line imaging modalities from both clinical and economic perspectives.

These findings suggest that a sequential imaging approach may not always be necessary, and FCH-PET should be reserved for select cases where conventional imaging is inconclusive. This study supports a personalized diagnostic strategy, where ultrasound and SPECT serve as initial screening tools, followed by FCH-PET or 4D-CT when required. These insights reinforce the need for optimized clinical guidelines, balancing diagnostic accuracy and cost-effectiveness in preoperative parathyroid localization.

Table 1 summarizes the characteristics and results of all papers included in this review.

## 4. Discussion

In recent years, a growing number of studies, many of which have been included in the present review, have confirmed the substantial interest this technique has generated in the localization of enlarged parathyroid glands.

A review from Park and Coll [41] emphasized that while ultrasonography (US) and 99mTc-sestamibi single-photon emission computed tomography/computed tomography (SPECT/CT) remain first-line imaging modalities, their limitations in multiglandular disease, ectopic glands, and recurrent disease necessitate the use of advanced imaging techniques. They reviewed the role of FCH-PET and 4D-CT, highlighting the increasing evidence supporting their use, particularly in challenging cases. FCH-PET showed superior sensitivity compared to SPECT/CT, and has a role in cases where conventional imaging is inconclusive. However, 4D-CT provides superior anatomical detail and remains valuable, especially in redo-surgery settings and ectopic gland localizations.

The authors also discuss the radiation burden of 4D-CT, which remains a concern despite its high diagnostic accuracy.

The authors advocate for a sequential imaging strategy, wherein US and SPECT/CT serve as first-line modalities, with FCH-PET and/or 4D-CT reserved for inconclusive cases. This aligns with emerging evidence supporting a multimodal approach tailored to individual patient profiles.

**Figure 1:**
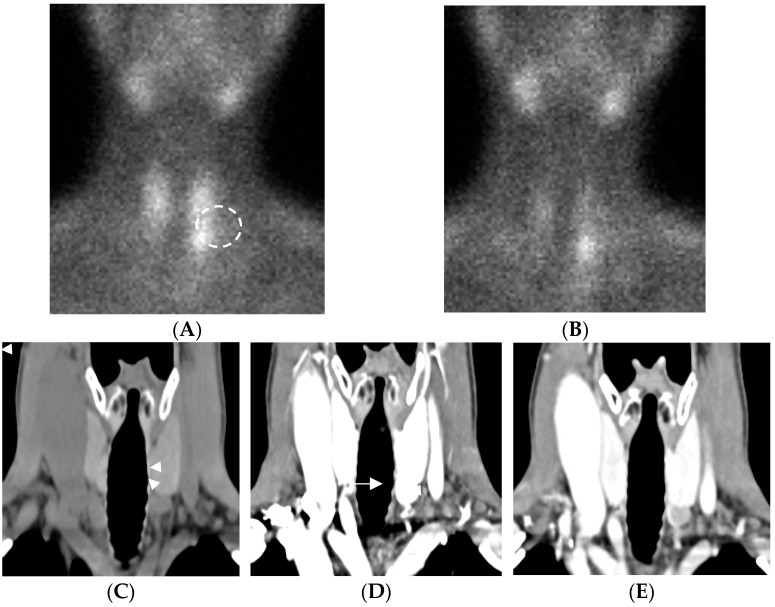
Left inferior parathyroid adenoma in a 50-year-old woman. (**A**) Scintigraphy with 99mTc-sestamibi: a nodular uptake (dashed circle) is observed on the left side, slightly more intense than the uptake in the adjacent superior portion of the thyroid lobe. (**B**) On the delayed scan, this focal lesion shows a clearly delayed washout compared to the surrounding thyroid tissue. 4D-CT coronal reformatted images: (**C**) hypodense nodule (arrowheads) on plane image with respect to thyroid gland, (**D**) enhancing on arterial phase, and (**E**) hypodense on venous phase with respect to thyroid gland. “Polar vessel sign” (arrow) is clearly visible. The 4D-CT scan shows no thyroid tissue around the parathyroid gland (no intrathyroidal parathyroid).

**Figure 2:**
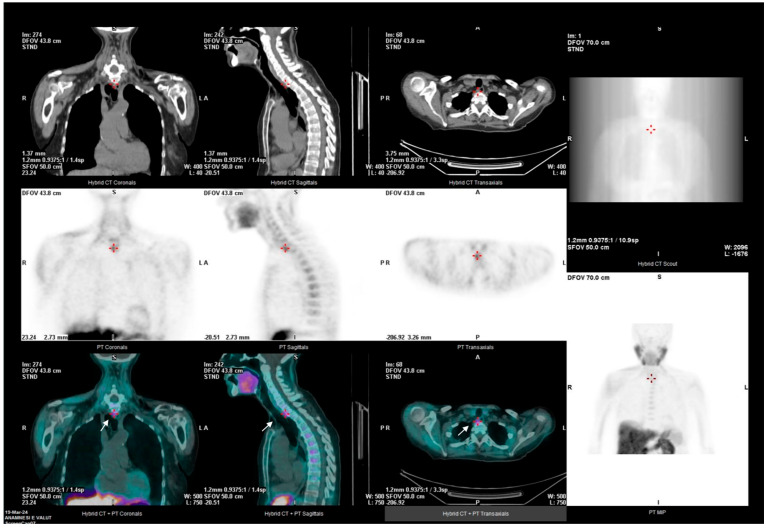
A 74-year-old woman diagnosed with normocalcemic primary hyperparathyroidism with bone complications. The 18F-fluorocholine PET/CT scan shows a posterior parathyroid, though it does not provide precise details regarding the origin of the localized gland.

**Figure 3:**
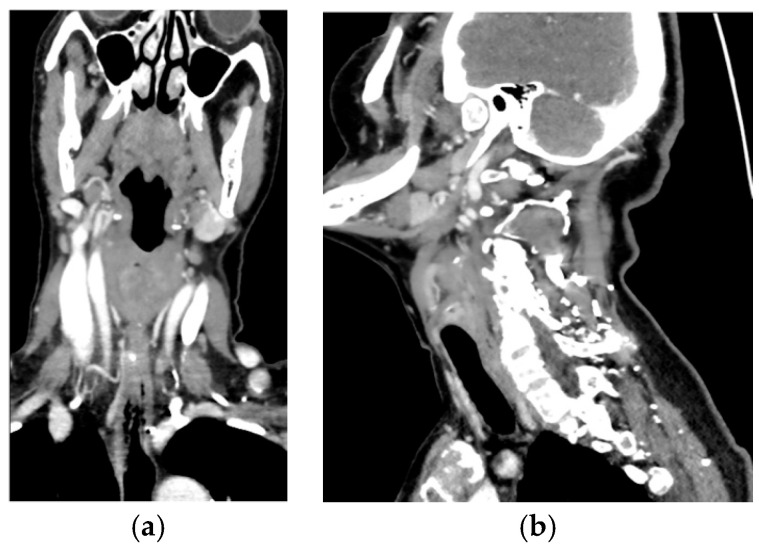
(**a**,**b**) The same case examined with 4D-CT shows the parathyroid pedicle originating from the upper cervical sections, confirming the embryological origin of the superior parathyroid (PIV). Moreover, 4D-CT has also proven to be very effective in the localization of the parathyroid of median retroesophageal location because it allows identification of the side of the vascular pedicle, facilitating the minimally invasive approach even in such circumstances. This is because this imaging technique can easily identify a parathyroid, even in an abnormal position, but it is not certain that this is really the pathological one because this is not functional imaging. Therefore, it is always necessary to combine it with MIBI scintigraphy or FCH-PET [42].

The role of 4D-CT appears complementary, particularly for anatomical localization in complex cases, as shown in Figure 1, Figure 2 and Figure 3.

When comparing the two methods, one must also consider cost, availability of each method, and exposure to ionizing radiation. While FCH-PET offers superior sensitivity and functional imaging capabilities—particularly in cases of inconclusive first-line diagnostics—it remains significantly more expensive and less widely available in many clinical settings. Conversely, 4D-CT, although anatomically precise and associated with lower overall costs, involves higher radiation exposure due to multiphase contrast imaging, albeit with a notably shorter acquisition time [7,16,40,42].

Recent improvements in 4D-CT protocols have significantly reduced radiation exposure, narrowing the gap with FCH-PET. Current estimates indicate an effective dose of approximately 6–10 mSv for 4D-CT and 4–8 mSv for FCH-PET, depending on equipment and protocol optimization [7,40,41]. Although radiation burden remains a consideration—particularly in younger patients—it is generally outweighed by diagnostic performance, availability, and cost in clinical decision-making.

In the present review, hereditary pHPT has not been mentioned. As there is often multigland parathyroid involvement, this category poses a challenge for parathyroid imaging. There are several hereditary forms of primary hyperparathyroidism (pHPT), including MEN1, MEN2A, MEN4, and hyperparathyroidism-jaw tumor syndrome (HPT-JT), in which imaging often proves non-contributory. Among these, MEN1 typically presents with a clinical phenotype similar to that of sporadic pHPT [43]

In familial forms, imaging is frequently negative, with reported detection rates in the literature as low as 29%. [44]. It is important to note that these hereditary syndromes usually involve all four parathyroid glands, which are often small and hyperplastic—features that may account for the low sensitivity of both anatomical and functional imaging techniques [45].

Historically, preoperative imaging was not routinely performed in MEN1 cases, as surgical management consisted of bilateral neck exploration. However, this approach was progressively reconsidered due to the risk of unrecognized ectopic localizations. Today, the same radiological and nuclear imaging protocols are recommended for both hereditary and sporadic PHPT.

Notably, the most recent guidelines from the French Society of Endocrinology recommend fluorocholine PET/CT as the first-line nuclear imaging modality in MEN1 patients. For these reasons, in the present work, we have deliberately focused on the sporadic forms of PHPT [43,45].

This review presents limitations that reflect the lack of prospective randomized trials on the subject. However, it can be of practical support, especially in concentrating ‘difficult’ cases in hospitals equipped with a full range of diagnostic tools and the necessary expertise for their correct use and interpretation.

From the information gathered, we can state that, actually, FCH-PEt is the method of choice for second-line investigation in the case of failure of first-line imaging in locating enlarged parathyroid glands. Its characteristics, along with the use of more advanced scanning techniques, have significantly enhanced the possibility of preoperative localization of enlarged parathyroid glands, which can then be treated with minimally invasive focused techniques, resulting in substantial reduction of operative time, minimized procedural aggressiveness, and containment of complications. However, the role of 4D-CT remains irreplaceable due to the anatomical details it provides, owing to the highest spatial resolution currently available among routine imaging modalities. Therefore, in the presence of low PTH values, very posterior glands, or suspicion of intra-thyroid localization of an enlarged parathyroid gland, the examination may follow or even replace the FCH-PET.

## 5. Conclusions

The preoperative localization of pathological parathyroid glands in primary hyperparathyroidism has significantly evolved with the advent of advanced imaging techniques such as FCH-PET and 4D-CT. The former offers excellent sensitivity, particularly in detecting small or ectopic adenomas, and is especially useful when first-line imaging modalities yield inconclusive results. Its high diagnostic performance has enhanced the feasibility of focused, minimally invasive parathyroid surgery.

Conversely, 4D-CT provides unmatched anatomical detail, making it an indispensable tool in complex or reoperative cases and in specific scenarios such as low PTH levels or suspected intrathyroidal localization.

Comparative studies support a sequential and personalized imaging strategy, balancing diagnostic efficacy, patient-specific factors, and cost considerations. While FCH-PET may emerge as the preferred second-line option in most clinical settings, 4D-CT retains a valuable complementary role. However, the current evidence base is limited by the lack of prospective randomized trials. Thus, a definitive, standardized diagnostic algorithm cannot yet be established. Nonetheless, the data available to date support a general framework that can guide clinicians in optimizing diagnostic pathways for individual patients.

## Figures and Tables

**Table 1:** Synoptic table of PET/CT and 4D-CT tr.

Study	Year	N° Patients	Sensitivity PET/CT (%)	Sensitivity 4D-CT (%)	Main Conclusion
Abbott [34]	2012	535	-	-	4D-CT improves localization and reduces bilateral explorations but increases costs.
Michaud [11]	2015	17	94	-	FCH-PET is superior to US and SPECT for pHPT localization.
Lundstroem [35]	2016	43	-	76	4D-CT is effective in patients with negative sestamibi scans.
Taywade [31]	2017	5	100	100	FCH-PET and 4D-CT show perfect concordance in adenoma localization.
Quak [13]	2018	25	91.3	-	FCH-PET is effective in cases with negative conventional imaging.
Zajickova [12]	2018	13	92	-	FCH-PET is useful for small glands and multiple adenomas.
Fischli [14]	2018	23	95.5	-	FCH-PET is highly accurate when other imaging is negative/equivocal.
Kluijfhout [17]	2019	44	94.3	-	PET/CT is beneficial after inconclusive conventional imaging.
Broos [15]	2019	271	96	-	FCH-PET shows a high detection rate and may be first-line imaging.
Xue [16]	2019	17	84.1	-	FCH-PET is more sensitive than SPECT and US in uremic hyperparathyroidism.
Mazurek [20]	2021	65	100	-	FCH-PET has 100% sensitivity in preoperative localization.
Dudoignon [19]	2022	51	76	-	FCH-PET is more accurate than conventional imaging and useful for complex cases.
Dekorsky [21]	2022	33	72.7	-	FCH-PET is effective in cases where conventional imaging was negative.
Manyalich-Blasi [27]	2022	37	92.1	-	FCH-PET is superior to US and SPECT.
Al-Difaie [36]	2023	60	-	88	4D-CT maintains high sensitivity even in patients with low PTH levels.
Goren [9]	2023	72	92.7	-	FCH-PET facilitates minimally invasive surgery with high cure rates.
Seydinia [29]	2023	321	99	-	Large retrospective study confirming FCH-PET as first-line functional imaging.
Van den Bruel [22]	2023	104	78.8	-	FCH-PET improves localization precision for superior vs. inferior adenomas.
Wolf [23]	2023	147	90	-	FCH-PET is superior in detecting small adenomas; useful as second-line imaging.
Battistella [38]	2023	296	99	86	FCH-PET is superior in selected cases with a sensitivity of 99%.
Hairston [37]	2024	437	-	81.2	4D-CT is superior to US for lateralization but both are useful.
Quak [24]	2024	57	82	-	Randomized trial shows FCH-PET superior to MIBI SPECT/CT for first-line use.
Kaseb [39]	2024	166	83	53	FCH-PET is superior, but adding 4D-CT improves accuracy in inconclusive cases.
Batora [40]	2024	5000	92	82	Decision-model analysis. 4D-CT is cost-effective; FCH-PET is more accurate but less cost-effective.

## References

[1] Bilezikian J.P., Bandeira L., Khan A., Cusano N.E. (2018). Hyperparathyroidism. Lancet.

[2] Gasparri G. (2017). Updates in Primary Hyperparathyroidism. Updates Surg..

[3] Gurrado A., Pasculli A., Avenia N., Bellantone R., Boniardi M., Merante Boschin I., Calò P.G., Camandona M., Cavallaro G., Cianchi F. (2023). Parathyroid Retrospective Analysis of Neoplasms Incidence (pTRANI Study): An Italian Multicenter Study on Parathyroid Carcinoma and Atypical Parathyroid Tumour. J. Clin. Med..

[4] Erickson L.A., Mete O., Juhlin C.C., Perren A., Gill A.J. (2022). Overview of the 2022 WHO Classification of Parathyroid Tumors. Endocr. Pathol..

[5] Del Rio P., Boniardi M., De Pasquale L., Docimo G., Iacobone M., Materazzi G., Medas F., Minuto M., Mullineris B., Polistena A. (2024). Management of surgical diseases of Primary Hyperparathyroidism: Indications of the United Italian Society of Endocrine Surgery (SIUEC). Updates Surg..

[6] Ovčariček P.P., Giovanella L., Hindie E., Huellner M.W., Talbot J.-N., Verburg F.A. (2022). An essential practice summary of the new EANM guidelines for parathyroid imaging. Q. J. Nucl. Med. Mol. Imaging.

[7] Hindié E., Schwartz P., Avram A.M., Imperiale A., Sebag F., Taïeb D. (2021). Primary Hyperparathyroidism: Defining the Appropriate Preoperative Imaging Algorithm. J. Nucl. Med..

[8] Cazaentre T., Clivaz F., Triponez F. (2014). False-Positive Result in 18F-Fluorocholine PET/CT Due to Incidental and Ectopic Parathyroid Hyperplasia. Clin. Nucl. Med..

[9] Goren S., Paladino N.C., Laks S., Cuny T., Vaillant-Lombard J., Mennetrey C., Assaf D., Hindié E., Guerin C., Fargette C. (2022). Diagnostic Rechallenge with 18F-FCH PET/CT Often Allows Minimally Invasive Parathyroidectomy While Maintaining Exceptional Cure Rates. World J. Surg..

[10] Jarabek L., Jamsek J., Cuderman A., Rep S., Hocevar M., Kocjan T., Jensterle M., Spiclin Z., Lezaic Z.M., Cvetko F. (2022). Detection and localization of hyperfunctioning parathyroid glands on [18F]fluorocholine PET/ CT using deep learning—model performance and comparison to human experts. Radiol. Oncol..

[11] Michaud L., Balogova S., Burgess A., Ohnona J., Huchet V., Kerrou K., Lefèvre M., Tassart M., Montravers F., Périé S. (2015). A Pilot Comparison of 18F-fluorocholine PET/CT, Ultrasonography and 123I/99mTc-sestaMIBI Dual-Phase Dual-Isotope Scintigraphy in the Preoperative Localization of Hyperfunctioning Parathyroid Glands in Primary or Secondary Hyperparathyroidism. Influence of Thyroid Anomalies. Medicine.

[12] Zajíčková K., Zogala D., Kubinyi J. (2018). Parathyroid Imaging by 18F-Fluorocholine PET/CT in Patients with Primary Hyperparathyroidism and Inconclusive Conventional Methods: Clinico-Pathological Correlations. J. Physiol. Res..

[13] Quak E., Blanchard D., Houdu B., Le Roux Y., Ciappuccini R., Lireux B., de Raucourt D., Grellard J.-M., Licaj I., Bardet S. (2018). F18-choline PET/CT guided surgery in primary hyperparathyroidism when ultrasound and MIBI SPECT/CT are negative or inconclusive: The APACH1 study. Eur. J. Nucl. Med. Mol. Imaging.

[14] Fischli S., Suter-Widmer I., Nguyen B.T., Müller W., Metzger J., Strobel K., Grünig H., Henzen C. (2018). The Significance of 18F-Fluorocholine-PET/CT as Localizing Imaging Technique in Patients with Primary Hyperparathyroidism and Negative Conventional Imaging. Front. Endocrinol..

[15] Broos W.A.M., Wondergem M., Knol R.J.J., van der Zant F.M. (2019). Parathyroid imaging with 18F-fluorocholine PET/CT as a first-line imaging modality in primary hyperparathyroidism: A retrospective cohort study. EJNMMI Res..

[16] Xue Y., Li W., Xia Z., Lei C., Cao Y., Wang Z., Pang H. (2019). The role of 18F-FCH PET/CT in patients with uremic hyperparathyroidism compared with 99mTc-sestaMIBI SPECT/CT and ultrasonography. EJNMMI Res..

[17] Kluijfhout W.P., Vorselaars W.M., van den Berk S.A., Vriens M.R., Borel Rinkes I.H., Valk G.D. (2016). Fluorine-18 fluorocholine PET-CT localizes hyperparathyroidism in patients with inconclusive conventional imaging: A multicenter study from the Netherlands. Nucl. Med. Commun..

[18] Evangelista L., Ravelli I., Magnani F., Iacobone M., Giraudo C., Camozzi V., Spimpolo A., Cecchin D. (2020). 18F-choline PET/CT and PET/MRI in primary and recurrent hyperparathyroidism: A systematic review of the literature. Ann. Nucl. Med..

[19] Dudoignon D., Delbot T., Cottereau A.S., Dechmi A., Bienvenu M., Koumakis E., Cormier C., Gaujoux S., Groussin L., Cochand-Priollet B. (2022). 18F-fluorocholine PET/CT and conventional imaging in primary hyperparathyroidism. Diagn. Interv. Imaging.

[20] Mazurek A., Dziuk M., Witkowska-Patena E., Chudzinski W., Piszczek S., Gizewska A., Saracyn M. (2022). The utility of 18F-fluorocholine PET/CT in the imaging of parathyroid adenomas. Endokrynol. Pol..

[21] Dekorsy F.J., Beyer L., Spitzweg C., Schmidmaier R., Todica A., Trupka A., Cyran C.C., Berger F., Ladurner R., Zimmermann P. (2022). Preoperative Imaging with [18F]-Fluorocholine PET/CT in Primary Hyperparathyroidism. J. Clin. Med..

[22] Van den Bruel A., Bijnens J., Van Haecke H., Vander Poorten V., Dick C., Vauterin T., De Geeter F. (2023). Preoperative imaging for hyperparathyroidism often takes upper parathyroid adenomas for lower adenomas. Sci. Rep..

[23] Wolf H.W., Nebiker C.A. (2023). Preoperative identification of small parathyroid adenomas—Better done by fluorocholine positron emission tomography/computed tomography. Gland. Surg..

[24] Quak E., Lasne-Cardon A., Cavarec M., Lireux B., Bastit V., Roudaut N., Salaun P.-Y., Keromnes N., Potard G., Vaduva P. (2024). F18-Choline PET/CT or MIBI SPECT/CT in the Surgical Management of Primary Hyperparathyroidism: A Diagnostic Randomized Clinical Trial. JAMA Otolaryngol. Head Neck Surg..

[25] Christensen J.W., Ismail A., Søndergaard S.B., Bennedbæk F.N., Nygaard B., Jensen L.T., Trolle W., Holst-Hahn C., Zerahn B., Kristensen B. (2022). Preoperative imaging in primary hyperparathyroidism: Are 11C-Choline PET/CT and 99mTc-MIBI/123Iodide subtraction SPECT/CT interchangeable or do they supplement each other?. Clin. Endocrinol..

[26] Quak E., Lasne Cardon A., Ciappuccini R., Lasnon C., Bastit V., Le Henaff V., Lireux B., Foucras G., Jaudet C., Berchi C. (2021). Upfront F18-choline PET/CT versus Tc99m-sestaMIBI SPECT/CT guided surgery in primary hyperparathyroidism: The randomized phase III diagnostic trial APACH2. BMC Endocr. Disord..

[27] Manyalich-Blasi M., Domínguez-Garijo P., Saavedra-Pérez D., Sánchez-Izquierdo N., Casanueva Eliceiry S., Perissinotti A., Porta M.M., de Hollanda A., Hanzu F.A., Serrat M.A.L.-B. (2022). Comparison of [18F]fluorocholine PET/CT with [99mTc]sestamibi and ultrasonography to detect parathyroid lesions in primary hyperparathyroidism: A prospective study. Gland. Surg..

[28] Ramalho D., Rocha G., Oliveira J.M., Oliveira M.J. (2022). Fluorine-18 Fluorocholine Positron Emission Tomography/Computed Tomography in Primary Hyperparathyroidism: A Case Report and Review of Literature. Cureus.

[29] Seyedinia S.S., Mirshahvalad S.A., Schweighofer-Zwink G., Hehenwarter L., Rendl G., Pirich C., Beheshti M. (2023). Evolving Role of [18F]Flurocholine PET/CT in Assessing Primary Hyperparathyroidism: Can It Be Considered the First-Line Functional Imaging Approach?. J. Clin. Med..

[30] Imperiale A., Bani J., Bottoni G., Latgé A., Heimburger C., Catrambone U., Vix M., Treglia G., Piccardo A. (2023). Does 18F-Fluorocholine PET/CT add value to positive parathyroid scintigraphy in the presurgical assessment of primary hyperparathyroidism?. Front. Med..

[31] Taywade S.K., Damle N.A., Behera A., Devasenathipathy K., Bal C., Tripathi M., Agarwa S., Tandon N., Chumber S., Seenu V. (2017). Comparison of 18F-Fluorocholine positron emission tomography/computed tomography and four-dimensional computed tomography in the preoperative localization of parathyroid adenomas-initial results. Indian J. Endocr. Metab..

[32] Rodgers S.E., Hunter G.J., Hamberg L.M., Schellingerhout D., Doherty D.B., Ayers G.D., Shapiro S.E., Edeiken B.S., Truong M.T., Evans D.B. (2006). Improved preoperative planning for directed parathyroidectomy with 4-dimensional computed tomography. Surgery.

[33] Cheung K., Wang T.S., Farrokhyar F., Roman S.A., Sosa J.A. (2012). A Meta-analysis of Preoperative Localization Techniques for Patients with Primary Hyperparathyroidism. Ann. Surg. Oncol..

[34] Abbott D.E., Cantor S.B., Grubbs E.G., Santora R., Gomez H.F., Evans D.B., Lee J.E., Vu T., Perrier N.D. (2012). Outcomes and economic analysis of routine preoperative 4-dimensional CT for surgical intervention in de novo primary hyperparathyroidism: Does clinical benefit justify the cost?. J. Am. Coll. Surg..

[35] Lundstroem A.K., Trolle W., Soerensen C.H., Myschetzky P.S. (2016). Preoperative localization of hyperfunctioning parathyroid glands with 4D-CT. Eur. Arch. Otorhinolaryngol..

[36] Al-Difaie Z., Scheepers M.H.M.C., Engelen S.M.E., Havekes B., Bouvy N.D., Postma A.A. (2023). Diagnostic Value of Four- Dimensional Dynamic Computed Tomography for Primary Hyperparathyroidism in Patients with Low Baseline Parathyroid Hormone Levels. Diagnostics.

[37] Hairston H., Gardner J.R., Gibson A.C., Wright C., Small M., King D., Fitzgerald R., Spencer H.J., Bodenner D.L. (2024). Four-dimensional computed tomography and ultrasonography for prediction of pathological parathyroid location: A retrospective review of a single surgeon’s patients at a single institution. Gland. Surg..

[38] Battistella E., Pomba L., Toniato R., Burei M., Gregianin M., Watutantrige Fernando Toniato A. (2023). Evolution of the Diagnosis and Treatment of Primary Hyperparathyroidism. J. Clin. Med..

[39] Kaseb A., Benider H., Treglia G., Cusumano C., Bessac D., Trimboli P., Vix M., Piccardo A., Latgé A., Imperiale A. (2025). Refining the role of presurgical PET/4D-CT in a large series of patients with primary hyperparathyroidism undergoing [18F] Fluorocholine PET/CT. Eur. J. Clin. Investig..

[40] Bátora D., Iskandar R., Gertsch J., Kaderli R.M. (2024). Impact of perioperative diagnostic tools on clinical outcomes and cost- effectiveness in parathyroid surgery: A decision model-based analysis. BMJ Open.

[41] Park H.S., Hong N., Jeong J.J., Yun M., Rhee Y. (2022). Update on Preoperative Parathyroid Localization in Primary Hyperparathyroidism. Endocrinol. Metab..

[42] Paladino N.C., Taïeb D., Sebag F. (2025). Parathyroid Imaging. Endocrine Surgery: Clinical Diagnosis and Management.

[43] Al-Salameh A., Haissaguerre M., Tresallet C., Kuczma P., Marciniak C., Cardot-Bauters C. (2025). Chapter 6: Syndromic primary hyperparathyroidism. Ann. Endocrinol..

[44] Gheorghe A.-M., Nistor C., Florescu A.-F., Carsote M. (2025). An Analysis of Primary Hyperparathyroidism in Individuals Diagnosed with Multiple Endocrine Neoplasia Type 2. Diseases.

[45] Schubert L., Gaillard M., Melot C., Delbot T., Cottereau A.S., Koumakis E., Bonnet-Serrano F., Groussin L. (2025). Management of primary hyperparathyroidism in MEN1: From initial subtotal surgery to complex treatment of the remaining gland. Ann. Endocrinol..

